# A Randomized Trial of Atropine vs Patching for Treatment of Moderate Amblyopia

**Published:** 2011-08-01

**Authors:** A R Medghalchi, S Dalili

**Affiliations:** 1 Department of Ophthalmology, Amiralmomenin Hospital, School of Medicine, Guilan University of Medical Sciences, Rasht, Iran; 2 Department of Pediatrics endocrinology, Nemazee Hospital, Shiraz University of Medical Sciences, Shiraz, Iran

**Keywords:** Moderate amblyopia, Patch theraphy, Atropine penalization

## Abstract

**Background:**

amblyopia is a major cause of visual impairment in children. Our aim is to compare patching and atropine penalization for treatment of moderate amblyopia in 4-10 years old children.

**Methods:**

During 2004-2007, in a randomized clinical trial, 120 patients aged 4-10 years old with moderate amblyopia in the range of 20/40 -20/100 were enrolled. Subjects randomized either to patch therapy or twice weekly atropine penalization in equal groups and were followed for 2 years. The success rate was defined as increment of 2 or more lines of visual acuity or final visual acuity of 20/25 or better.

**Results:**

The visual acuity in amblyopic eye improved from base line to a mean of 3.8 lines in patching group and mean of 3.7 lines in atropine group at the end of follow up. The average visual acuity in both groups was 0.5 Log MAR acuity that increased to 0.18 in patching group and 0.2 in atropine group.

**Conclusion:**

Twice weekly atropine penalization could improve visual acuity of a magnitude like to improvement provided by patching in treatment of moderate amblyopia in patients aged 4-10 years

## Introduction

Amblyopia is the most common cause of monocular visual loss in children.[1] Patch and atropine penalization of the sound eye has been the mainstay of the sound eye.[2][3][4] Simons et al.,[5] reported that atropine therapy (one drop in a week) was a successful treatment as daily therapy. A pediatric eye disease investigation group6 reported that 2 hours patching improved the moderate to severe amblyopia in [3][4][5][6][7] years children. Pediatric Eye Disease Investigation Group reported that daily atropine or patching has similar improvement of moderate amblyopia.[2][3][4] We conducted a randomized clinical trial to compare the success rate of atropine administration twice a week with 2 hours patching for treatment of moderate amblyopia.

## Materials and Methods

This prospective randomized clinical trial was undertaken in 120 subjects. Patients with the following criteria were enrolled.[1]. Age of 4-10 years, [2]. Visual acuity (VA) in amblyopic eye between 20/40 and 20/100, [3]. Difference of visual acuity between eyes of equal or more than 3 Log MAR, [4]. Difference in refractive error between eyes of equal or more than 1 diopter for hyperopia and 1.5 diopter for astigmatism,

[5]. Wearing of optical correction for at least 4 weeks and 6. At least 2 years follow-up.

Informed consent was obtained from the subjects before treatment. Randomization was done on the patients records, the even number assigned to 3 hours patch therapy and odd numbers for those undergone penalization with 0.5% atropine twice a week. The number of subjects in each group was 60 patients.

Cycloplegic refraction was done with 0.5% cyclopentolate. Suitable glass was prescribed and after one month of wearing of glass, VA was checked with E chart (nidek projector) at initiation of treatment and at each visit.

Successful treatment was defined as 2 or more lines of improvement in VA or VA of 20/25 or better in amblyopic eye. Then final mean of VA or amblyopic eye and rate of successful treatment of both groups were evaluated with SPSS software (Version 16, Chicago, IL, USA). This study was approved by Ethics Committee of Guilan University of Medical Sciences.

## Results

Between January 2004 and January 2007, 120 subjects entered this study. Sixty patients were assigned to patching group and the same assigned to penalization group. About 45% of patients were female and the remainders were male. The mean VA in patching and penalization groups at initiation of treatment were

0.46 and 0.45 Log MAR respectively. The pre-treatment data of patients were summarized in Table 1.

**Table 1:** Pre treatment patients’ data

****		**Total patients**	**Patching group**	**Penalization group**
Sex	Male	55%	54%	56%
	Female	45%	46%	46%
Age	4-<6	50	26	24
	6-<8	50	24	26
	8-10	20	10	10
VA in amblyopia				
Snellen	Log MAR			
20/100	0.7	15	8	7
20/80	0.6	17	8	9
20/60	0.48	31	15	16
20/50	0.4	25	13	12
20/40	0.3	32	16	16
Mean Log MAR		0.45	0.45	0.45
VA in sound eye				
Snellen	Log MAR			
20/20	0	80	41	39
20/25	0.1	20	9	11
20/30	0.2	15	7	8
20/40	0.3	5	3	2
Amount of anisometropia				
1-<2		60	31	29
2-<3		30	15	15
3-<4		20	9	11
4-<5		10	5	5

Sixty subjects were enrolled including patching group, so that in patients with two lines acuity difference between eyes, 2 hours patch therapy was performed and in subjects with three or more lines difference, 3 hours patch therapy was started. The successful rate of treatment was about 76%. About 50% of patients achieved VA of 20/25 or more at the end of follow-up. The frequency of patients achieved special level of VA in both groups that was summarized in Table 2. The mean increment of visual from base line was 3.6 lines. About 35% of achieved stereo acuity of 400 second of arc at the end of follow-up. The VA and improvement at the end of two years were summarized in Table 2.

**Table 2:** Frequency of patients developed specific level of vision at the end of two years

****		**Patching**	**Penalization**	**Total **
Number of patients		60	60	120
VA								
Snellen	Log MAR			
>20/100	<0.7	100%	100%	100%
>20/80	<0.6	100%	100%	100%
>20/60	<0.48	99%	97%	98%
>20/50	<0.4	95%	93%	94%
>20/40	<0.3	88%	88%	88%
>20/30	<0.2	74%	76%	75%
>20/25	<0.1	51%	49%	50%
>20/200	0	25%	25%	25%
Mean Log MAR		0.15	0.17	0.16

Sixty subjects received penalization with 0.5% atropine twice a week. During improvement of the VA, the penalization dose decreased to one drop weekly. The successful rate of treatment was about 74%. About 50% of patients achieved VA of 20/25 or more at the end of follow-up. About 30% of patients achieved stereoacuity of 400 second of arc at the end of follow-up.

Among 120 patients, VA test at the end of treatment in both groups was summarized. The successful rate of treatment was about 75%. About 50% of patients achieved VA of 20/25 or more at the end of follow-up. The frequency of patients achieved the special level of VA in both groups was summarized in Table 2.

## Discussion

We compared the effects of patching and atropine penalization for treatment of moderate amblyopia in 120 subjects aged 4-10 years in a randomized clinical trial. The mean VA at the end of treatment was 0.18 Log MAR or 20/30 in both groups. The VA in amblyopic eye increased from base line to a mean of 3.8 lines in patching and 3.6 lines in penalization groups. There was no statistically significant difference between both groups. The successful rate of treatment in penalization and patching groups were 74% and 76% respectively. There was no significant difference between the 2 groups in mean of VA or line of improvement. About 35% of patching group and 30% of atropine group achieved stereoacuity of 400 second of arc at the end of follow-up. Pediatric Eye Disease Investigation Group[3] in their randomized trial of atropine VS patching of moderate amblyopia reported that 51% of patching group and 49% of atropine group achieved VA of 20/25 or better. Also at the end of follow-up, VA improved from base line to a mean of 3.7 lines in the patching and 3.6 lines in atropine group. PEDIG[7] in a randomized trial of atropine VS patching for treatment of amblyopia for follow-up at age of 10 years showed that patching and penalization with atropine resulted in a comparable improvement in VA of amblyopic eye. Their reports were like our results. PEDIG[2] in another study showed that visual acuity improved in both groups from base line 3.16 lines in patch group and 2.84 lines in atropine group. The success rates of treatment were 79% in patching group and 74% in atropine group. Simons[5] reported that atropine treatment may produce better binocular function than patching tr eatment.

Our study showed that patching treatment and penalization with atropine had the same therapeutic effect for treatment of moderate amblyopia in patients of 4-10 years old.
